# An experimental investigation of the convective heat transfer augmentation in U-bend double pipe heat exchanger using water-MgO-Cmc fluid

**DOI:** 10.1038/s41598-024-63043-6

**Published:** 2024-05-30

**Authors:** Mustafa M. Gabir, Ibrahim M. Albayati, Mohammad Hatami, Dhirgham Alkhafaji

**Affiliations:** 1 Department of Mechanical Engineering, University of Babylon, Hillah, Babylon Iraq; 2 Air Conditioning and Refrigeration Techniques Engineering Department, Al-Mustaqbal University College, Hillah, Babil 51001 Iraq; 3 School of Engineering, University of Lincoln, Brayford Way, Lincoln, LN6 7TS UK; 4 Department of Mechanical Engineering, Esfarayen University of Technology, Esfarayen, North Khorasan Iran

**Keywords:** Heat exchanger, Double pipe U-bend, Magnesium oxide, Carboxymethyl cellulose, Heat transfer enhancement, Convection, Chemical engineering, Mechanical engineering, Other nanotechnology, Renewable energy

## Abstract

One of the major problems of using nanofluids in heat exchange applications is the forming and deposition of nanoparticles on the inner surface of the heat exchanger. In this paper, Water-Cmc fluid is used as a surfactant for nanoparticles to prevent deposition and congregation. The pressure drops and heat transfer in U-bend double pipe heat exchanger based on water-MgO-Cmc fluid, are examined. Nanoparticles of Magnesium Oxide (MgO) and Carboxymethyl Cellulose (Cmc) are used with pure water as a base fluid. The experimental rig and procedures are designed to facilitate various operational conditions such as flow rate, volume concentration of MgO particles and weight concentration of Cmc particles. Furthermore, convective heat transfer coefficient, heat exchanger effectiveness, pressure drop, friction factor, under different conditions, are measured. The results demonstrate convective heat transfer coefficient of U-bend double pipe heat exchangers is enhanced by 35% for 1 MgO vol.% and 0.2 Cmc wt.% compared to base fluid (Water-Cmc). It is concluded that pressure drops are directly proportion to the increase of MgO nanoparticles at same Cmc concentration by 23% at 0.2 wt.%. Whilst, friction factor of the system is inversely proportion to the increase of volumetric flow rate of water-MgO-Cmc fluid. An increase in MgO nanoparticle concentration increases the friction factor, hence maximum friction factor enhancement by 38% for MgO concentration of 1 vol.%. The effectiveness of heat exchanger is slightly increased by 8% with increase of MgO concentration and flow rate. Finally, thermo-physical characteristics of water-MgO-Cmc fluid at various temperatures, are measured

## Introduction

**Figure 1:**
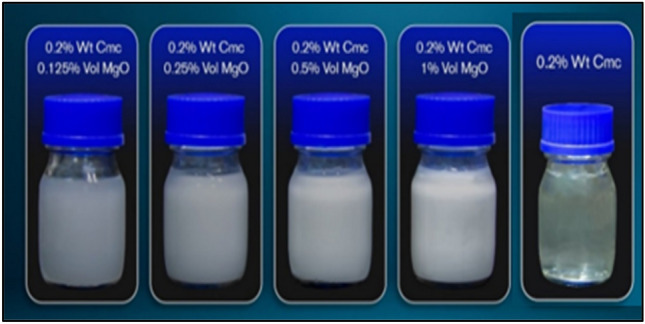
Sample of the water-MgO-Cmc fluid.

One of the major problems of using nanofluids in heat exchange applications is the forming and deposition of nanoparticles on the inner surface of the manifold and pipes, which significantly reduces the efficiency of the heat exchanger [ref]. Heat exchangers play major role in different industrial fields like food processing, oil and gas, refrigeration and district cooling, steel manufacturing, petrochemical, and many other industrial applications. The required amount of heat transfer has been achieved by using multi-working fluid through sufficient contact of heat exchanger surface area. Thereby, multi types of heat exchangers are being utilized in industrial sectors depending upon required amount of heat transfer and the specifications and configuration requirement of the thermal devices^1,2^. The heat exchanger performance improves by the increase in the thermal conductivity of working fluid during heat exchange process. The working fluid flows through inner pipes of the double pipe heat exchanger and transfer heat via forced convection mode. The enhancement in the convection coefficient is mainly dependent upon enhancement in nanofluid thermal conductivity. Several research in this field reported that improving the convection coefficient will lead to improve of nanofluids thermal conductivity^3–6^. The diameter of nano particles as small as 10 nm is recommended in many nanofluid research. However, nanoparticles diameter below 100 nm is commonly adopted in most of the published research^7^. It is very important to demonstrate thermal performance of systems under effect of single and two phases flow^8–17^. Improvement of the thermo-physical properties is realized to be the main way of improving efficiency of the heat exchangers operated by passive technique^18–28^. Mahrooghi and Moghiman^7^ demonstrated nanofluid application in cooling systems, in addition to the applications of the nano-fluids in thermal engineering systems. Several studies shown that the enhancement in thermo-physical characteristics like thermal conductivity and density of working fluid by adding different types of nanoparticles to base fluid, resulting further pressure drop and decrease in specific heat capacity. Nanoparticles accumulate and deposit at the bottom of the container is another challenge associated using nanofluids. Moreover, uneven distribution of particles in the base fluid leads to unstable thermo-physical characteristics of the nanofluid [ref]. Han et al.^29^ implemented several sets of experimentations to investigate effect of using alumina aqueous nanofluid as a working fluid in the double pipe heat exchanger at various bulk temperatures, on the heat exchange performance. Using various volumetric concentration of nano particles 0.25 and 0.5% to form nanofluid. Bahraei et al.^30^ investigated a new non-Newtonian hybrid nanofluid to improve hydraulic and thermal characteristics of double tube mini-channel heat exchangers. They have utilized 2 nano-fluids, which are, Gum Arabic (GA) coated Carbon Nano-tubes and Tetra Methyl Ammonium Hydroxide coated Fe_3_O_4_ NPs. Results demonstrated that the thermal exchange rate was improved by the addition of nanoparticles at lower values of Reynolds number. At Reynolds numbers 500 and 2000 of nanofluid, the thermal exchange rate enhanced by 54% and 29%, respectively, in compression with pure water. Vinod et al.^31^ improved performance of helical and shell coil heat exchangers through utilizing 3 different non-Newtonian nano-fluids, were dispersion of nanoparticles (Al_2_O_3_ CuO and Fe_2_O_3_) in an aqueous carboxymethyl cellulose (Cmc) base fluid at concentrations weight ranges from 0.2 to 1.0%. The non-Newtonian nano-fluid and pure water have been implemented on the shell and tube sides, respectively. The results addressed several parameters effected heat exchanger performance, like nano-fluid concentration, shell side temperature, and flow rate. It has been found that when these parameters increased, the Nusselt number would increase, Also the increase in stirrer speeds cause an increase in Nusselt number, resulting an improvement in heat exchange rate. Kumar et al.^32^ scrutinized the use of two nanofluid types, which are CeO_2_-water and ZnO-water in plate heat exchanger. A comparison was made of thermal exchange performance of this heat exchanger when utilizing nanofluid or pure water. The nanoparticles added to pure water by volume concentrations 0.50 to 2%. Results showed that the better thermal exchange performance may be obtained with the use of the ZnO-water nano-fluid as working fluid, the hot volumetric flow rate was 2 lpm and the cooled volumetric flow rate ranges from 0.5.0 to 2.0 lpm. The temperature of the inlet hot and cool fluids were 50 and 25 °C, respectively. It has been reported that max enhancement accrued at high nanoparticles concentrations and flow rates. Furthermore, many studies showed that the increase in nanoparticles concentrations led to the reductions in levels of thermal performance of thermal system. For instance, Zhang et al.^33^ conducted experimental study by presenting high heat flux heat exchanger utilizing graphene oxide–water nano-fluid at weight concentration range 0–0.05%. The results demonstrated undermined thermal performance of the heat exchanger under effects of single and two phases fluid flow. The major reason of the deteriorated convection heat transfer coefficient had been due to conglomerated and of nanoparticles in the bottom of tank, this led to the poor distribution of heat exchange and instability of the nanofluid. Same problem was also reported by Das et al.^34^ about natural convection and swimming pool Boiling heat transfer of the liquid-alumina nanoparticles. The experiments were investigated by alumina nanoparticles volume concentration ranges from 1 to 4% which dispersed in pure water. The impairment loss in heat transfer exchange has been seen in convective mode and boiling place. Anoop et al.^35^ presented deterioration in heat exchanger’s thermal performance. Experiments that have been conducted by using silicon dioxide–water nanofluids at weight concentrations 2, 4, and 6% as working fluid. Results from these experiments are compared versus results of using pure water as working fluid. It had been shown the pressure drop increased when nanoparticles weight concentrations increased. It can be concluded from previous published literature that the applications of nanofluid have limited usage due to conglomerated and deposition of nanoparticles in container, which leads to poor heat exchange. Therefore, in this paper, the work will include preparation of the water-MgO-Cmc fluid and the measurement of thermo-physical characteristics of this fluid. MgO nanoparticles utilized in system which need high pressure flow. An experimental research will be carried out to demonstrate the effect of variation flow rate and particles concentrations on the performance of heat exchanger, convection heat transfer coefficient, overall heat transfer coefficient, friction factor, and pressure drop of heat exchanger, will be measured. Cmc has been selected as dispersion media for MgO nanoparticles, it is used as a good dispersant for MgO nanoparticles by preventing them from agglomerating and dispersing at the bottom of the container, as well as providing stability for the base fluid. Using Cmc as surfactant, nanofluid used in heat exchanger to enhance heat transfer rate better than base fluid. Nanoparticles that dissolved in water suffer from agglomeration and deposition problems, hence, affect the circulation of the nanofluid, this will reduce heat exchange rate. The agglomeration and deposition problems could be solved by using Cmc as surfactant as shown in Fig. 1 and to enhance thermophysical process.

## Testing and experimental preparation

### Testing rig

**Figure 2:**
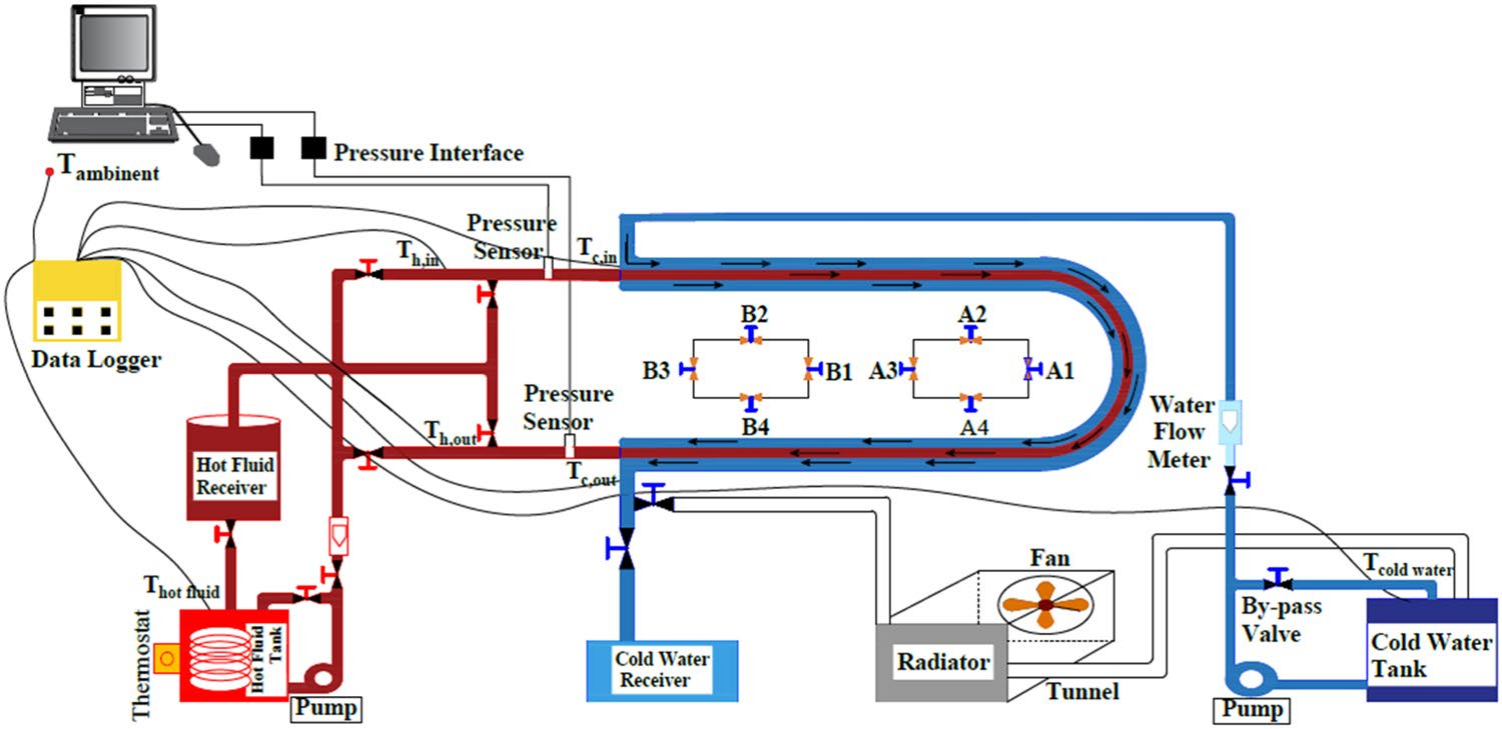
Diagram of the testing design rig.

The experimental setup of testing rig which is implemented in this research is illustrated in Fig. 2. This rig builds up from six major components sections, cold fluid loop, hot fluid loop, U-bend double pipe test section, temperature and pressure sensors, and data acquisition instruments. There are two centrifugal pumps manufactured by Shimge Company used one for cold fluid loop and another for hot fluid loop. Hot (water-MgO-Cmc) fluid loaded in insulation tank and return to accumulation tank to reduce pressure flow of hot fluid and turbulence in fluid layers. The cold fluid (pure water) loaded in another tank and circulated to radiator to cool it down and return it to another accumulation tank. Table 1 presented accuracy of instruments that used in the preparing the rig.

**Table 1:** Accuracy of instruments.

No	Instruments	Accuracy
1	Thermocouples Type K for temperature range (− 50.1 to 100 °C)	± (0.4% + 1 °C)
2	Pressure transducer Piezo resistive transmitter	± 0.5%
3	Interface system	± 0.2%
4	Pressure regulator	± 0.25%
5	Air flow meter	± 0.2%
6	liquid flow meter	± 1%

The applied temperature of hot fluid is set at 50 °C and volumetric flow rate varies from 8 to 14 L/min, while the cold fluid temperature set at 25 °C and constant volumetric flow rate 24 L/min. The temperature of the cold and hot fluids is measured by the use of the thermostats. Two rotameters are used for measuring flow rates, one for hot nano-fluid and the second for cold fluid. Temperatures of tanks and outlet and inlet of cold and hot fluid measured by 12 channels data logger Model BTM-4208SD manufactured by LUTRON ELECTRONIC. The pressure of nanofluid at inlet and outlet of inner pipe measured by pressure transducer manufactured by KELLER model PR-23R /80710-34. All rotameters, thermocouples, temperature data logger and pressure transducer were calibrated by The Central Agency for Standardization and Quality Control—Iraq. The test section of experimental setup is based on U-bend double-pipe heat exchanger consisting of 2 pipes, first copper pipe with a 540 cm length and inner and outer diameters of 20 mm and 22 mm, respectively. Second pipe is made from Perspex with a length of 500 cm and inner and outer diameter of 45 and 50 mm, respectively. Pure water and nanofluid flow in the annular side and inner pipes, respectively. The flow that has been examined in parallel flow contact within double pipe heat exchange. Experiments have been conducted at various flow rates, various volume fraction of MgO nanoparticles.

### Preparation of Water-MgO-Cmc fluid

To prepare water-MgO-Cmc fluid, two main steps were followed. Firstly, prepare the water-Cmc fluid, and secondly adding MgO nanoparticles. First step to prepare water-Cmc fluid, before adding Cmc powder, the pure water is heated up to approximately 40 °C. Thermo-physical characteristics of the pure water present in Table 2 for various temperatures. The hot pure water poured in a mixture container using a swirl device without stopping. The amount of Cmc that has been specified according to concentration range was added to pure water in very small quantities. Swirl device is kept on until finish adding the specified amount of Cmc powder. in order to ensure no agglomeration and accumulation of Cmc powder in the pure water. At the end of mixing operation all amount of the Cmc-water fluid placed in ultrasonic device (JTS-1018) for 8 h to complete mixing process and to ensure complete dissolution of Cmc powder in pure water. Ultra-sonic cleaner contained 12 transducers, basket and drain valve. The transducers wero to convert low frequency electrical signal (50 Hz) to high frequency (40 kHz) mechanical vibrations. The properties of carboxymethyl cellulose (Cmc) are listed in Table 3.

**Table 2:** Thermo-physical water properties^37^.

Temperature °C	Density (kg/m^3^)	Viscosity (kg/m s) × 10^–3^	Specific heat (J/kg K)	Thermal conductivity (W/m K)
25	997.1	0.8095	4183	0.5948
50	988	0.5042	4181	0.6305

**Table 3:** Properties for carboxymethyl cellulose (Cmc)^36^.

Material	ρ (kg/m^3^)	C_p_ (J/kg °C)	k (W/m °C)	µ (kg/m s) × 10^–3^
Cmc	1020	4.4	0.7	0.25

The amount of Cmc powder that added to pure water has been estimated with the use of law of mixtures in terms of weight concentration percentage^36^. The mass of Cmc of 0.2% concentration by weigh for 30 litter pure water was 60 g. This quantity calculated by Eq. (1) [ref].

1 $$\varphi =\frac{{m}_{Cmc}}{{m}_{Cmc}+ {m}_{w}}$$

where, m_Cmc_ is mass of Cmc particles in (g) and m_w_ is the mass of pure water in (g). Second step is to prepare the Water-MgO-Cmc fluid, this step done by adding MgO nanoparticles mean diameter less than 50 nm to the Cmc-water fluid. Adding nanoparticles’ weight to thirty liters of Water-Cmc based on the following equation reported by^38^ as:

2 $$\varphi \% =\frac{Volume \,of \,MgO}{Volume \,of \,MgO+ Volume \,of \,Water-Cmc}$$

3 $$\varphi \% =\frac{(\frac{{m}_{p}}{{\rho }_{p}})}{(\frac{{m}_{p}}{{\rho }_{p}})+ (\frac{{m}_{bf}}{{\rho }_{bf} })}$$

where, m_p_ is mass of MgO NPs, ρ_p_ is density of MgO nanoparticles, m_bf_ represents mass of Water-Cmc fluid and ρ_bf_ is density of the Water-Cmc fluid. In this research, three volume concentrations of nanoparticles are used as, (0.125%, 0.25%, 0.5% and 1%). The mass of every concentration that has been estimated from Eq. (3) as can be seen in Table 4 [ref]. The thermal properties of MgO particles in Table 5 [ref].

**Table 4:** Mass of the concentration added to thirty liters of pure water.

φ%	0.125%	0.25%	0.5%	1%
Mass (g)	136.05	272.44	546.25	1098.02

**Table 5:** Properties for MgO Nanoparticle^39^.

Material	ρ (kg/m^3^)	C_p_ (J/kg °C)	k (W/m °C)	µ (kg/m s) × 10^–3^
MgO	3580	955	48.4	0

**Figure 3:**
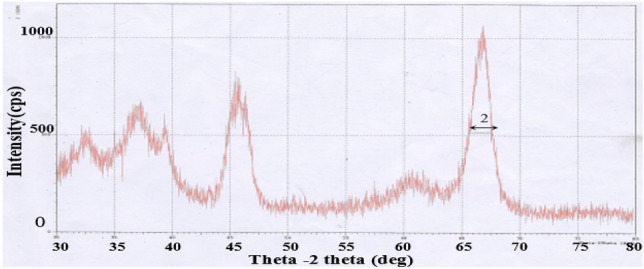
XRD pattern of MgO nanoparticles.

To ensure that MgO nanoparticle is less than 50 nm, a most commonly utilized X Ray Diffraction method (XRD) is adopted for estimating the average size of nanoparticles. Average nanoparticle size is found to be between 43 to 50 nm and full width at half maximum (FWHM) is taken from XRD pattern as presented in Fig. 3,.

### Thermophysical properties of water-MgO-Cmc fluid

**Figure 4:**
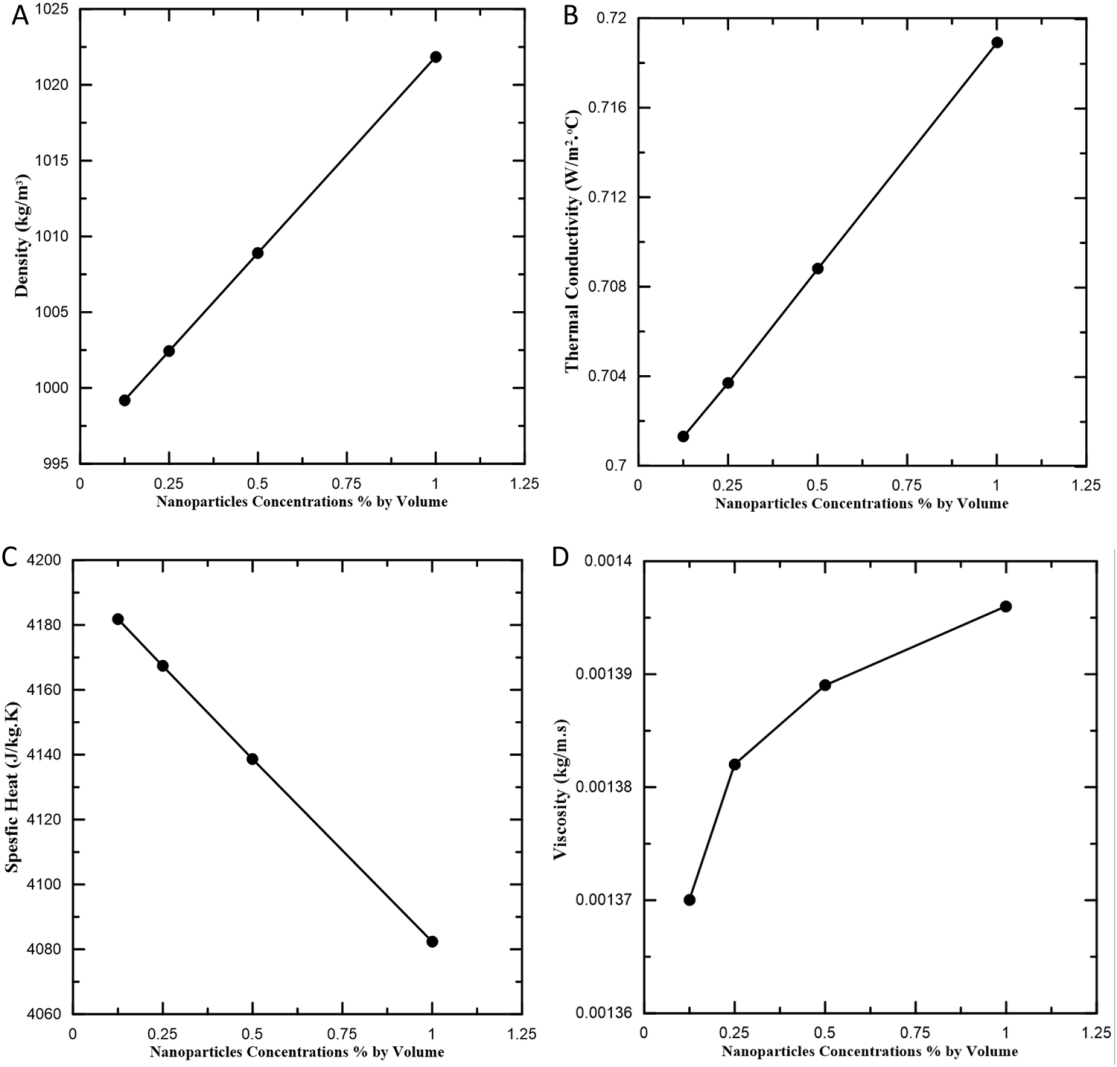
Experimental thermo-physical properties of the Water-MgO-Cmc fluid.

Thermo-physical characteristics of water-MgO-Cmc fluid are measured experimentally with suitable instruments for each concentration. Thermal conductivity and Density increase with increases in mass concentration of MgO particles (see Fig. 4a,b, respectively). However, heat capacity (Fig. 4c) decreases significantly with increasing mass concentration of MgO particles, while viscosity slightly increased with the increase in mass concentration of MgO particles (Fig. 4d). It is clear that the preparation of this fluid is accomplished in two stages:

1. First stage includes preparing the CMC-water fluid by adding CMC powder concentrations to distilled water normally at range 0.2% by weight.
2. Second stage includes adding MgO nanoparticles to the CMC-water fluid. The resulting solution from this addition is prepared in the same manner as shown in the “Preparation of water-MgO-Cmc fluid”. The concentration of MgO nanoparticles at (0.125%, 0.25%, 0. 5% and 1%) by volume. At this stage, the thermal properties will be measured in experimentally.

## Convection heat and heat transfer coefficients of the system

Heat transfer from hot (water-MgO-Cmc) and received by cold water are calculated^38^ as given in Eqs. (4) and (5). Equation (6) presents the average of heat transfer between two fluids.

4 $$Q_{h} = \mathop m\limits^{ \circ }_{h} \times C_{p.h} \times \left( {T_{h.in} - T_{h.out} } \right)$$

5 $$Q_{C} = \mathop m\limits^{ \circ }_{C} \times C_{p.c} \times \left( {T_{c.out} - T_{c.in} } \right)$$

where, Q_c_ is amount of heat for cold fluid, Q_h_ is amount of heat for hot fluid, Cp_c_ represents specific heat of cold fluid, T_c,in_ represents cold fluid temperature at inlet and T_c,out_ represents cold fluid temperature at outlet.

6 $${Q}_{ave}= \frac{{Q}_{c}+ {Q}_{h}}{2}$$

where, Q_ave_ represents mean amount of heat transfer between two fluids. The logarithmic mean temperature deference (LMTD) for the parallel arrangement of the flow^38^ is equal to.

7 $${LMTD}_{PF}=\frac{{\Delta T}_{1}-{\Delta T}_{2}}{ln[{\Delta T}_{1}/{\Delta T}_{2}]}$$

where: $${\Delta T}_{1}={T}_{h.in}-{T}_{c.in} ; {\Delta T}_{2}={T}_{h.out}-{T}_{c.out}$$

For counter flow arrangement of flow is equal to:

8 $${LMTD}_{CF}=\frac{{\Delta T}_{1}-{\Delta T}_{2}}{ln[{\Delta T}_{1}/{\Delta T}_{2}]}$$

where: $${\Delta T}_{1}={T}_{h.in}- {T}_{c.out} ;$$ $${\Delta T}_{2}={T}_{h.out}-{T}_{c.in}$$

The overall heat transfer coefficient for the annular side (U_o_), is given in Eq. (9)^38^.

9 $${U}_{o}=\frac{ {Q}_{ave}}{{A}_{o}LMTD}$$

Overall heat transfer coefficient for the inner pipe (U_i_), has been given in Eq. (10)^38^.

10 $${U}_{i}=\frac{ {Q}_{ave}}{{A}_{i}LMTD}$$

The heat exchange for the dual tube heat exchangers is estimated based on Eq. (11), without taking into account the term fouling factor^38^:

11 $$\frac{1}{{U}_{i }{A}_{i}}=\left[\frac{1}{{h}_{o}{A}_{o}}\right]+\left[\frac{lnln ( \frac{{D}_{0}}{Di}) }{2 \pi {k}L}\right]+\left[\frac{1}{{h}_{i}{A}_{i}}\right]$$

where, ${U}_{o}$, ${U}_{i}$ is the combined annular side and inner pipe side overall heat transfer coefficients and k represents thermal conductivity of pipe material. heat exchanger length is L (m)^39^.

12 $$N{u}_{0}= \frac{\left( \frac{f}{2}\right)\left(Re-1000 \right) Pr}{1.07+12.7 {\left(\frac{f}{2}\right)}^{0.5} ({Pr }^\frac{2}{3}-1 )}$$

13 $$f = (1.58lnln \left( {Re} \right) 3.82)^{ - 2} 2300{ } < Re < \,10^{6} ,{ }0.5\, < \,Pr < { }2000$$

where, the outer pipe (annular-side) heat transfer coefficient is calculated using Eq. (14)^39^:

14 $${h}_{o}= \frac{{N{u}_{0}\times k}_{o} }{ {D}_{h}}$$

where, ${D}_{h}$ is the hydraulic diameter, and is calculated as in Eq. (15)^39^, and ${k}_{o}$ is the outer pipe's thermal conductivity (annular-side)

15 $${D}_{h}= \frac{4A}{p}=( {D}_{0}-{D}_{i} )$$

The value of Nusselt number is obtained from Eq. (16). Equation (11) uses the ho value from Eq. (14) to calculate the inner pipe (tube side) heat transfer coefficient (${h }_{i}$ or ${h}_{nf}$), which represents sole unknown number in the equation. Reynolds number depends upon the flow rate at the pipe's inlet^40^.

16 $${Nu}_{nf}= \frac{{h}_{nf} \times {D}_{i}}{{k}_{nf}}$$

17 $${Re}_{nf}=\frac{{\rho }_{nf}v{D}_{i}}{{\mu }_{nf}}$$

The Prandtl number is determined by the thermal conductivity, viscosity, and specific heat of nanofluids at mean fluid temperature as given in Eq. (18)^40^

18 $${Pr}_{nf}=\frac{{cp}_{nf}{\mu }_{nf}}{{k}_{nf}}$$

Experimental friction factor has been determined based upon the differences in the value of the pressure between inlet and outlet of the pipe, as given in Eq. (19)^40^ and the expression is provided below:

19 $$f = \frac{\Delta P}{{\frac{{L_{{\dot{I}}} }}{{D_{i} }} \times \left( {\frac{{ v^{2} \rho_{nf } }}{2}} \right)}}$$

where: $\Delta P$ (pressure drop) = ${P}_{1}-{P}_{2}$

The effectiveness of NTU method is determined by Transfer unit's number of Eq. (20)^38^:

20 $$NTU = \frac{U \times A}{{C_{min} }} \Rightarrow NTU = \frac{Q}{{\left( {\Delta T} \right)_{LMTD} \times C_{min} }}$$

Heat capacity of the fluid in inner pipe is given in Eq. (21)^38^:

21 $$C_{h } = \mathop m\limits^{ \circ }_{h} \times C_{p.h}$$

Heat capacity of the fluid in annular side is given in Eq. (22)^38^:

22 $$C_{C } = \mathop m\limits^{ \circ }_{C} \times C_{p.C}$$

where ${C}_{min}$ is smaller than ${C}_{h}$ and ${C}_{C}$. The effectiveness can be calculated using Eq. (23)^38^

23 $$\varepsilon =\frac{1- exp [-NTU \left(1-Z\right)]}{1-Z exp [-NTU \left(1-Z\right)]}$$

24 $$\text{Where}: Z= \frac{{C}_{min}}{{C}_{max}}$$

## Results and discussion

Effects of the several operating parameters like volumetric flow rate and MgO nanoparticles mass concentration on U-bend double pipe heat exchanger performance are experimentally investigated. Effects of those parameters on convective heat transfer coefficient, heat exchanger effectiveness, friction coefficient and pressure drop, have been examined.

### Convection heat transfer coefficient

**Figure 5:**
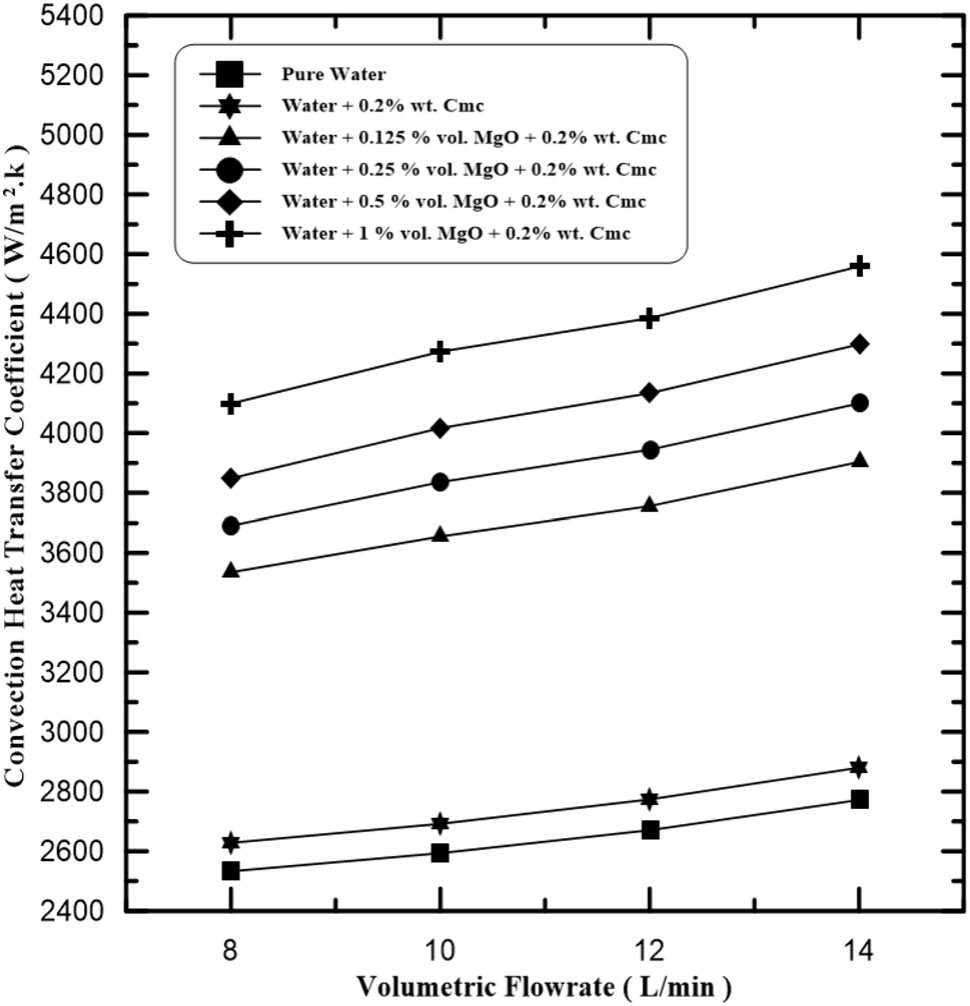
Convection coefficient at various MgO concentrations.

**Figure 6:**
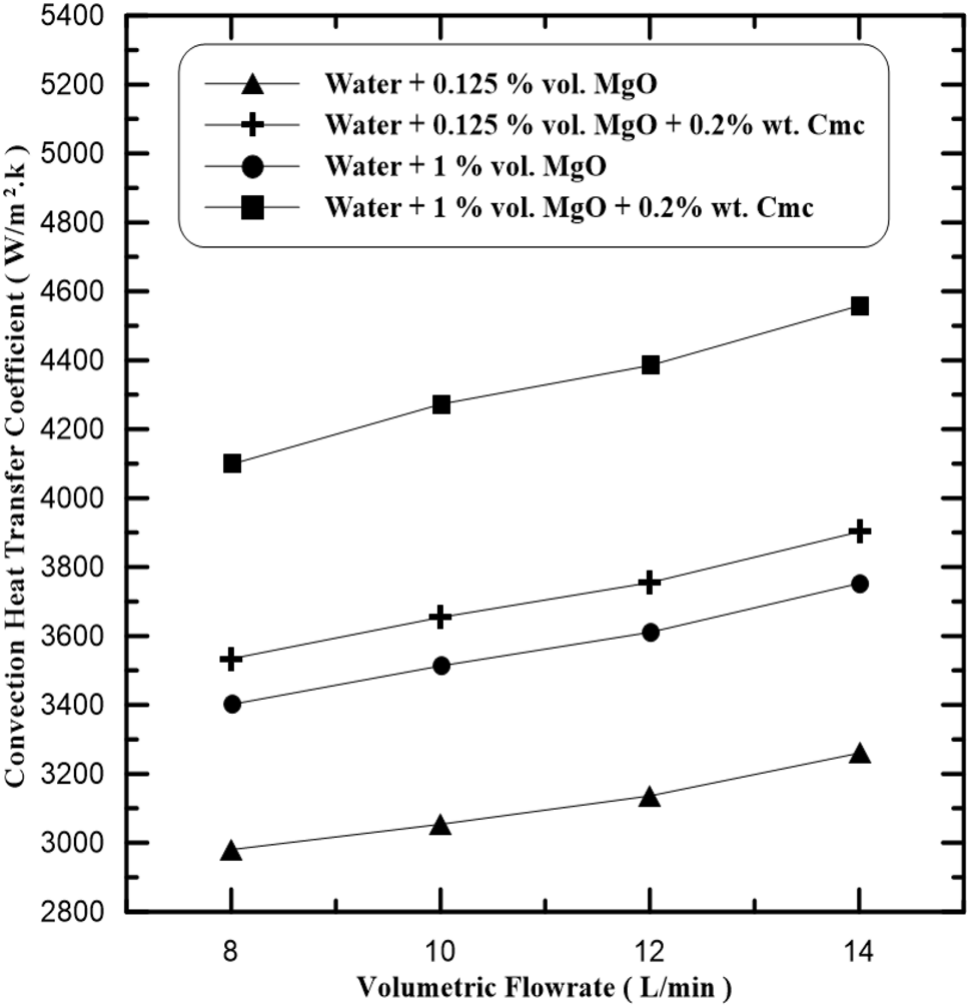
Compression of convection coefficient with and without Cmc addition.

The convection heat transfer coefficient increased for nano-fluid in comparison to the base fluid because of the increase in Brownian motion of nanoparticles, resulting an increase in the thermal conductivity of the entire system. The increase in volume concentration leads to increase in the thermal conductivity and viscosity but decrease in specific heat. Water-MgO-Cmc fluid is used as hot fluid flow in inner pipe value with volume flow rates range from (8, 10, 12 and 14 L/min), are as shown in Fig. 5, which presents the relation between the convection heat transfer coefficient of the Water-MgO-Cmc fluid with the flow rate of the fluid, for the pure water, water + 0.2% Cmc, and water + 0.2% Cmc + different volume concentrations of MgO NPs (0.125%, 0.25%, 0.5% and 1%). It is notable that the convection heat transfer coefficient of tested fluid increases as flow rates and concentration of MgO increase. Also, Fig. 6 present same relation and behavior of convection coefficient at same flow rate by make compression between cases of adding Cmc and cases of not adding. It is clear that heat convection coefficient values improved after adding 0.2% wt. Cmc from 3535.2 to 4100.21 at MgO concentrations 0.125% and 1%, respectively.

### The heat transfer effectiveness

**Figure 7:**
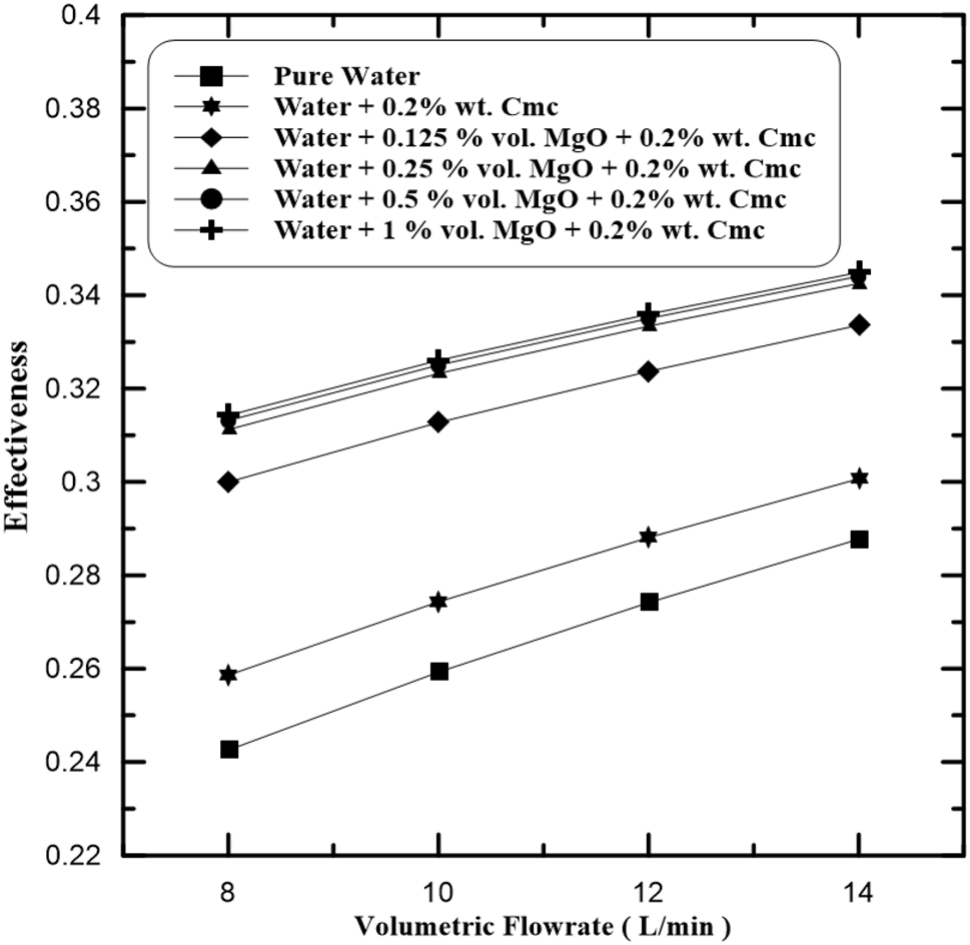
Heat exchanger effectiveness at various MgO concentrations.

**Figure 8:**
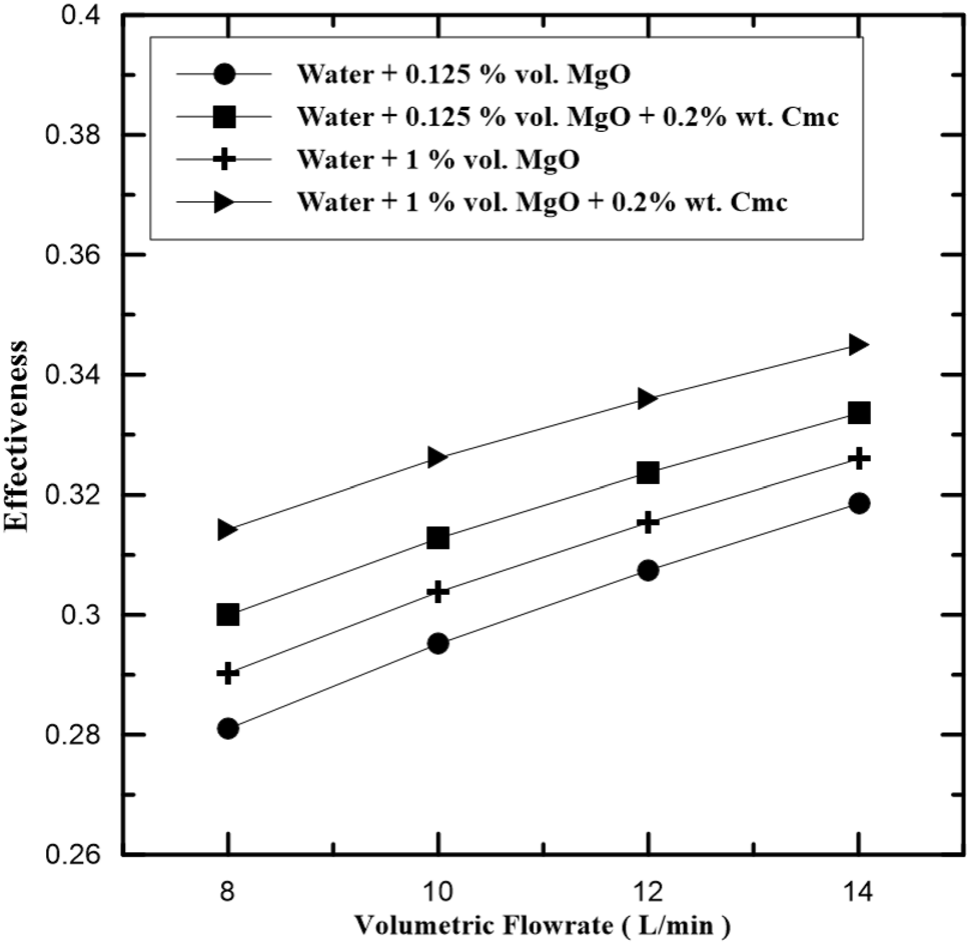
Compression of heat exchanger effectiveness with and without Cmc addition.

The Heat Transfer Effectiveness of double pipe heat exchangers using Water-MgO-Cmc Fluid is as presented in Fig. 7 illustrates the relationship of the heat exchanger effectiveness with hot fluid (Water-MgO-Cmc Fluid) volume flow rate in inner pipe at same conditions in previous section. It has been shown that the heat transfer effectiveness increases at increase MgO particles mass concentrations in base fluid (water-Cmc). So, the increase of flow rates of hot water from 8 to 14 L/min will increase average heat transfer which increases heat transfer effectiveness. The heat exchanger effectiveness slightly increases dependent on MgO concentrations in the Water-Cmc fluid. The concentrations of Cmc particles used was 0.2% by weight, and the MgO concentrations by volume changed at range of (0.125%, 0.25%, 0.5% and 1%). Figure 8 presents a compression of heat exchanger effectiveness in cases when using Cmc as a dispersant and cases of not using it. The highest values of effectiveness marks with 1% MgO by volume and 0.2% Cmc by weight as present. The effectiveness increases when add Cmc to the water-MgO fluid. when MgO concentrations 0.125% and 1% the effectiveness values 0.281 and 0.29, and these values enhance to 0.3 and 0.314, respectively. the previous values increase when flow rate of fluids increase.

### Pressure drop

**Figure 9:**
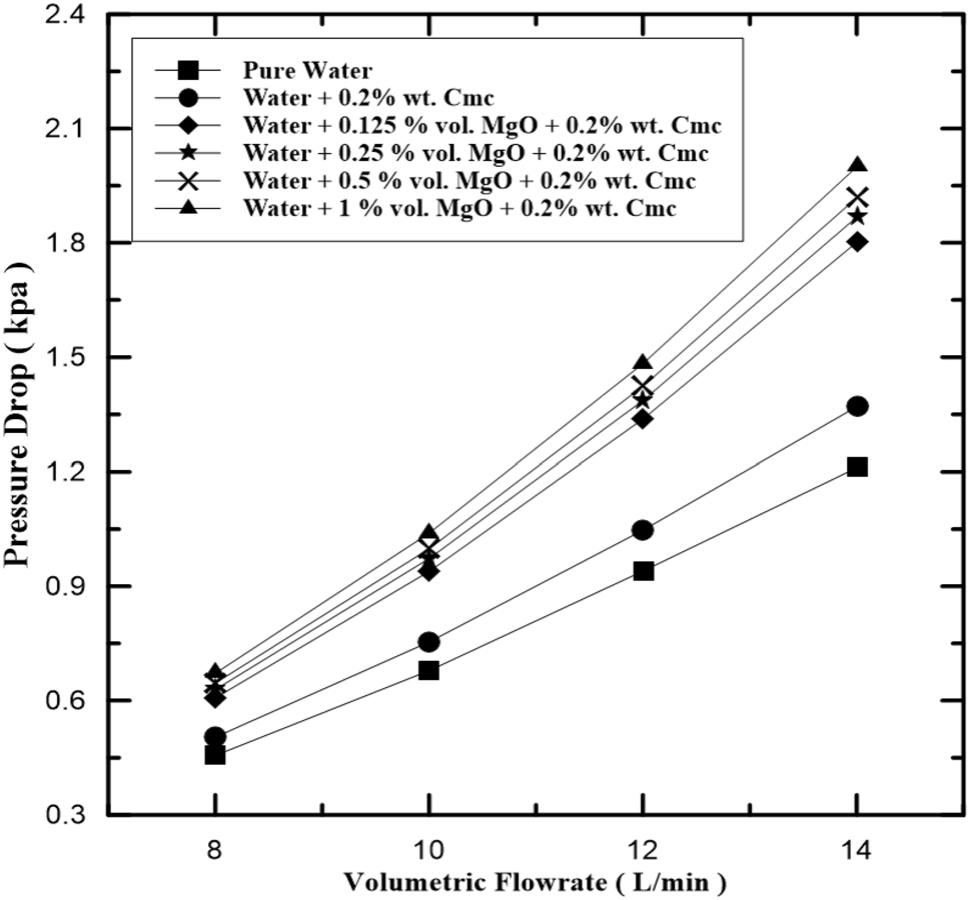
Pressure drop in inner pipe of heat exchanger at various MgO concentrations.

**Figure 10:**
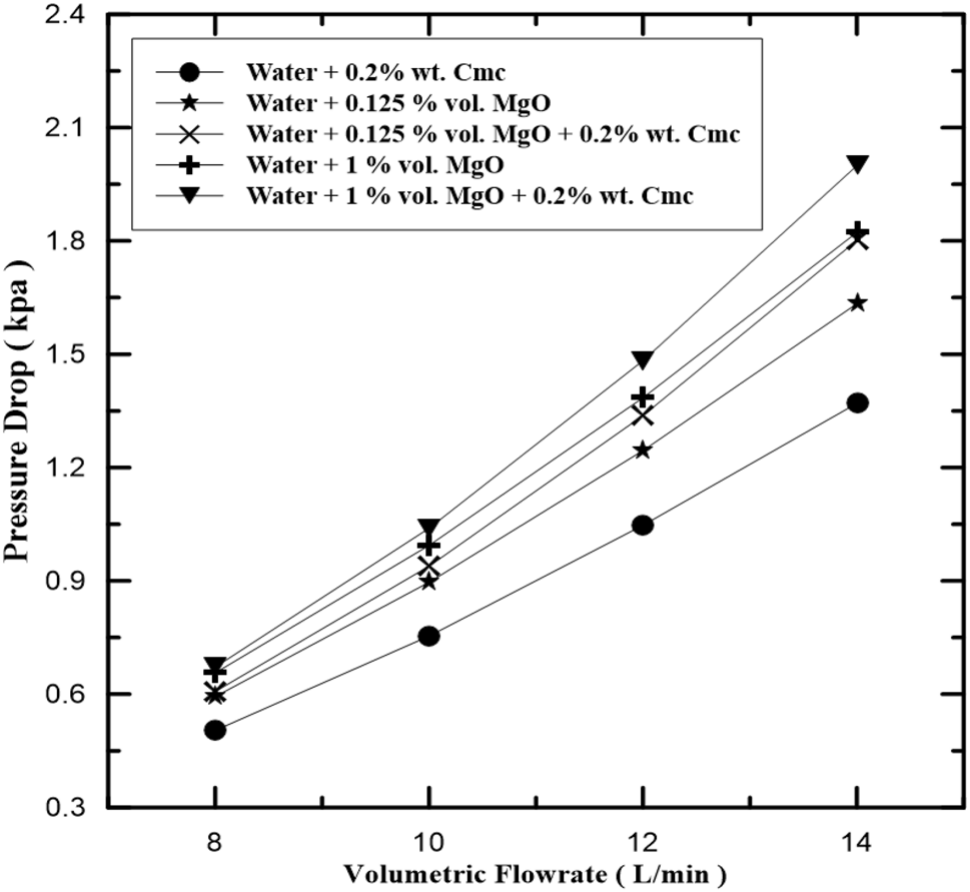
Pressure drop in inner pipe of heat exchanger with and without Cmc addition.

The relation between pressure drops and volumetric flow rate of hot fluid in inner pipe and pure water, the water-Cmc fluid, and the water-MgO-Cmc fluid, are shown in Fig. 9. It is clear that pressure has dropped due to the increase in the volumetric rate. It is found out that for the same volume flow rate that the level of pressure drops increases with nano-fluid concentration, this is because when the volume flow rate and the concentration of nanofluid increase pressure drop increases, this is because of the increased nanofluid viscosity and density, also the increase rate of collisions between the nanofluid particles and the Cmc particles and wall of pipe will obstruct the fluid flow. It is found that pressure drop for water + 1% vol. MgO + 0.2% wt. Cmc at maximum volume flow rate is the highest. The MgO particles disperse in the water- Cmc fluid and this gave stability to MgO particles and this led to increase pressure drop and enhance heat exchange rate. Figure 10 pointed on compression pressure drop in fluid with Cmc additives to nanofluid and another fluid without Cmc additives.

### Friction coefficient

**Figure 11:**
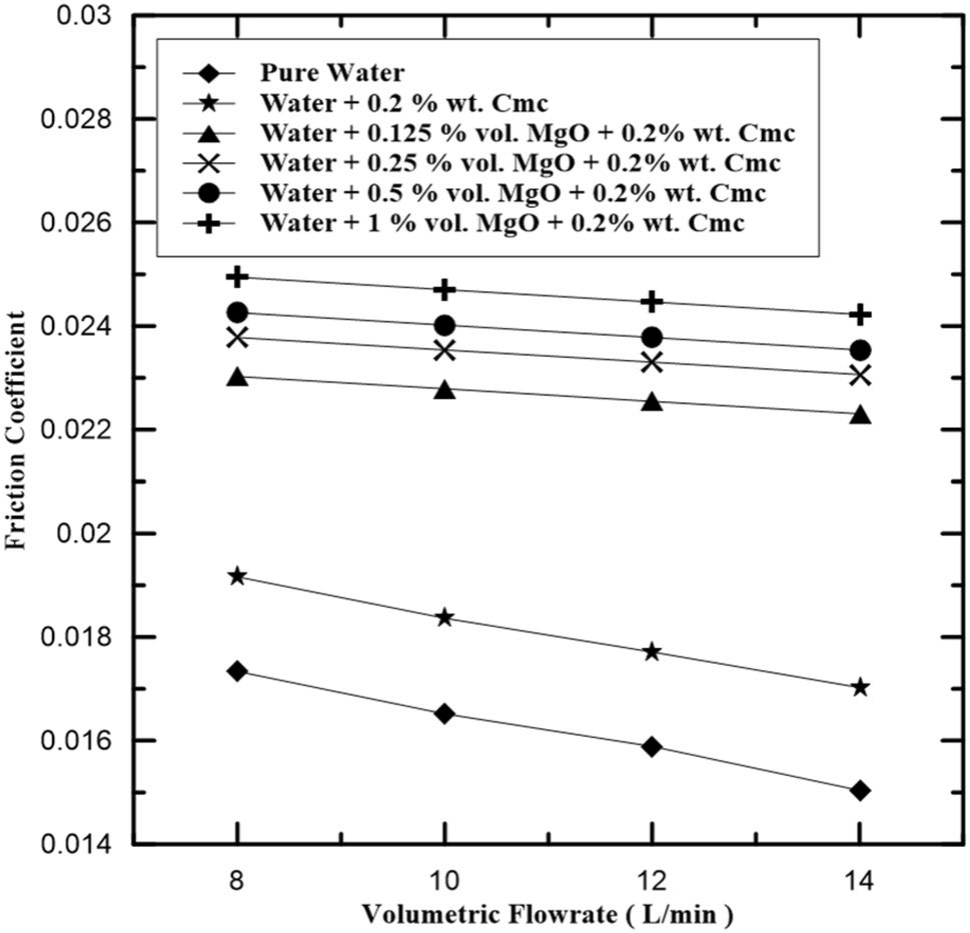
Friction coefficient in inner pipe of heat exchanger at various MgO concentrations.

**Figure 12:**
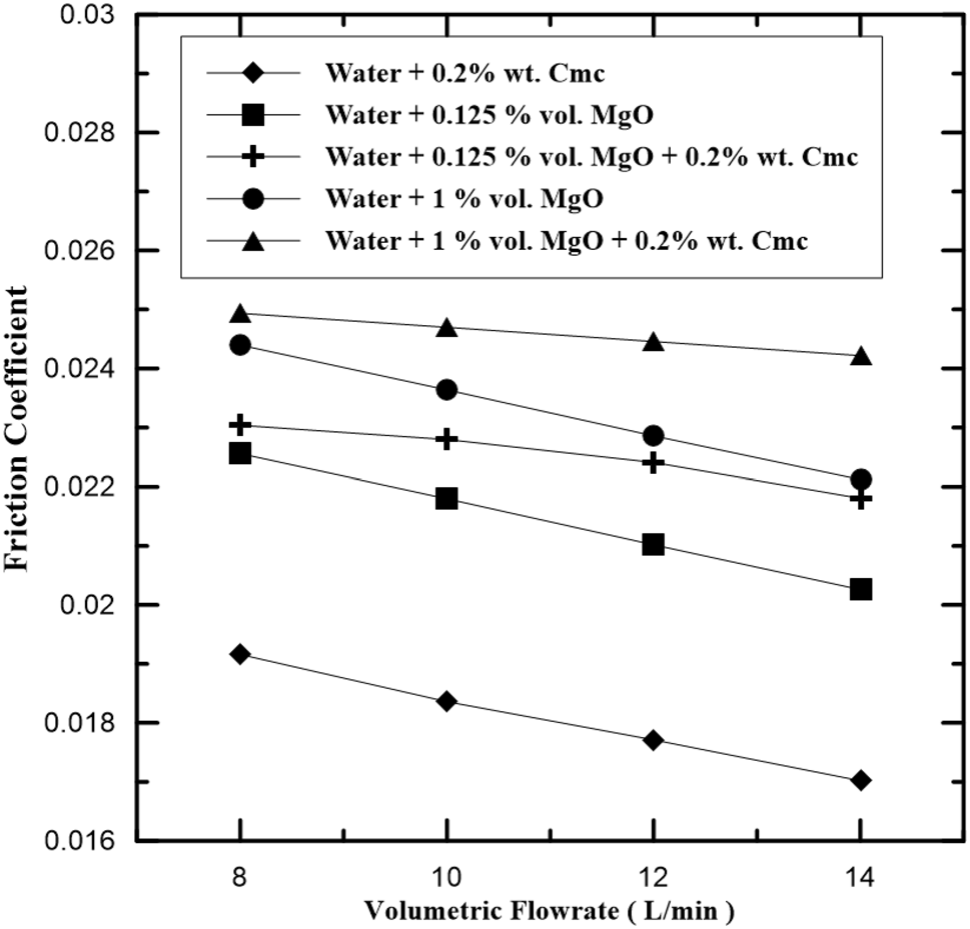
Friction coefficient in inner pipe of heat exchanger with and without Cmc addition.

**Figure 13:**
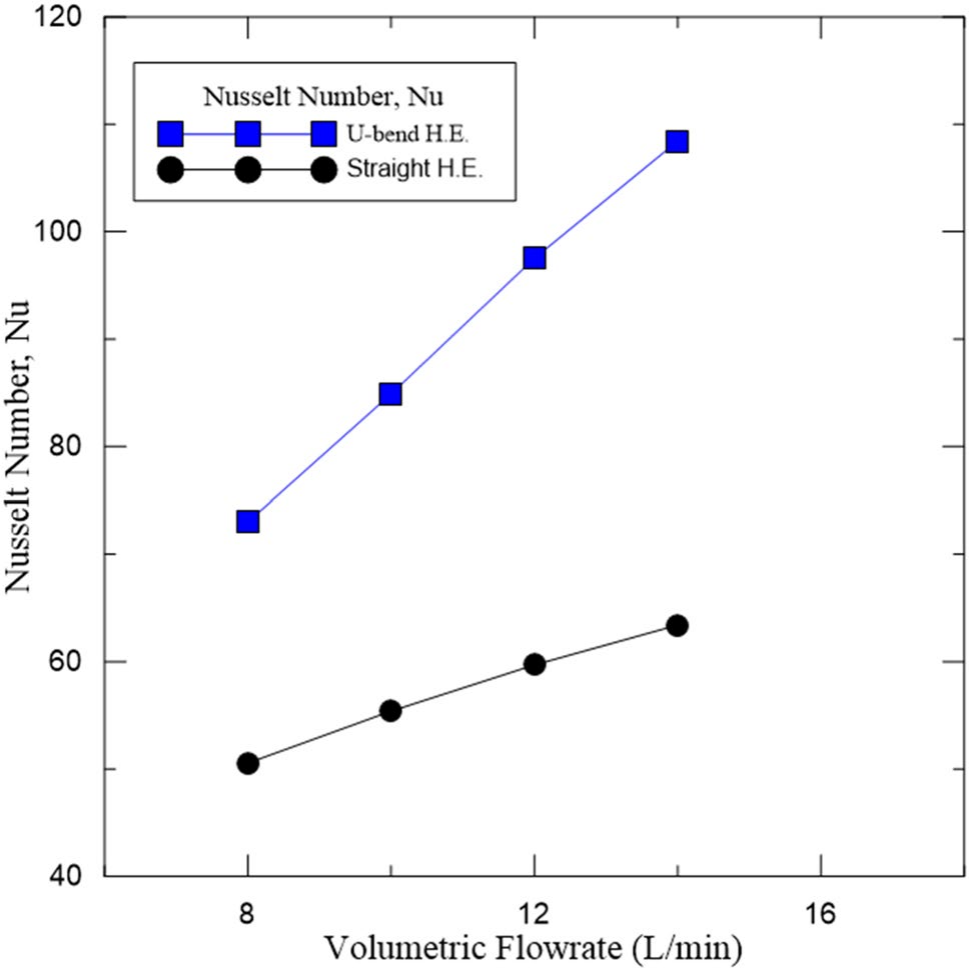
Presented compression performance of U-bend with straight heat exchanger. The improvement rate was approximately 36%.

The relation of friction coefficients for different hot fluid types with their volume flow rate, are presented in Fig. 11. It can be seen from Fig. 11 that the friction coefficients tend to decrease as volume flow rate increases according to Eq. (19). Figure 12 presents compression friction factor values when added Cmc at concentrations 0.2% wt., and this led to increase pressure drop and heat exchange rate because enhancement in physical properties for the water-Cmc fluid. the effect of added MgO nanoparticles at different concentrations present in Fig. 11 under various flow conditions and concentrations range (0.125%, 0.25%, 0.5% and 1% by volume). Generally, the fraction factor increases with the increase in concentration of MgO nanoparticle and Cmc that is due to increasing density of nanofluid (Fig. 13).

### compression study between U-bend and straight heat exchanger

## Conclusion

A double pipe heat exchanger rig has been built in order to determine heat transfer coefficient and pressure drop under effect of using Cmc as dispersant. The type of additives (Cmc and MgO) to pure water has an important impact on thermal conductivity enhancement which leads to improving heat transfer rates. It was concluded that thermal conductivity improved for Cmc concentrations 0.2% weight about (3%) and for MgO particles concentrations 1% volume about (5.5%). The enhancement in thermal conductivity at added concentrations MgO 1% volume and Cmc concentrations 0.2% weight about (6%), which presents a better case. The increase in concentrations of additives (Cmc and MgO) enhances viscosity of new fluid, which leads to increase pressure drop and friction coefficient. MgO nanofluid and Cmc particles will not result of a penalty drop in pressure but a little increase in the pressure drop when compared to pure water, so there is no need for additional pumping power. The nanofluid (Water-MgO) suffers from conglomeration and deposition in nanofluid tank. So, to prevent this problem Cmc is used as a surfactant for MgO particles in nanofluid. It was concluded, that MgO particles have high stability in Water-Cmc fluid and offer better heat exchange rate. So, the Water-MgO-Cmc fluid is considered as a better case, that shows more enhancement of heat transfer than the other fluids. Experimental results showed that heat transfer coefficient (h) is increased by added Cmc and the hot fluid volume flow rate. Heat transfer coefficient enhances about 5%. The heat transfer coefficient of Water-MgO-Cmc fluid increased with the increase of MgO particles concentrations. The heat transfer coefficient was enhanced by 25% when added 1% of MgO particles to pure water. Experimental results showed that heat transfer coefficient (h) when added Cmc and MgO is higher than other cases (pure water, water-Cmc and water-MgO) about (38%, 35% and 17%) respectively. It can be concluded that the effectiveness of heat exchanger (ε) is increased under effect of added Cmc as surfactant for nano fluid and enhanced by 23%. Also, pressure drop of water-MgO-Cmc fluid is higher than other cases by (30–40%).

## Data Availability

The datasets used and/or analyzed during the current study available from the corresponding author on reasonable request.

## References

[1] Hassaan AM (2022). An experimental investigation for the use of multi-wall carbon nanotubes based on water nanofluid in a plate heat exchanger. Heat Transf. Res..

[2] Hassaan AM (2023). Comparing the performance of using nanofluids in two different types of heat exchangers with the same heat transfer area. Heat Transf. Res..

[3] Hassaan AM (2022). An investigation for the performance of the using of nanofluids in shell and tube heat exchanger. Int. J. Therm. Sci..

[4] Hassaan, A. An investigation for the performance of the using of nanofluids in shell and tube. Available at SSRN 4001385 (2022).

[5] Hassaan AM (2022). Evaluation for the performance of heat transfer process in a double pipe heat exchanger using nanofluids. Proc. Inst. Mech. Eng. Part E J. Process Mech. Eng..

[6] Hassaan AM (2023). Experimental investigation of the performance of the plate heat exchanger using (multi-walled carbon nanotubes–Al_2_O_3_/water) hybrid nanofluid. Proc. Inst. Mech. Eng. Part E J. Process Mech. Eng..

[7] Mahrooghi A, Moghiman M (2015). Effect of nano particles on heat transfer in heat exchangers. Natura.

[8] Arya A (2017). Cooling of high heat flux flat surface with nanofluid assisted convective loop experimental assessment. Arch. Mech. Eng..

[9] Nakhjavani M (2017). green synthesis of silver nanoparticles using green tea leaves: Experimental study on the morphological, rheological and antibacterial behavior. Heat Mass Transf..

[10] Sarafraz M (2016). Thermal performance and viscosity of biologically produced silver/coconut oil nanofluids. Chem. Biochem. Eng. Q..

[11] Sarafraz M (2017). Low-frequency vibration for fouling mitigation and intensification of thermal performance of a plate heat exchanger working with CuO/water nanofluid. Appl. Therm. Eng..

[12] Sarafraz M (2018). Thermal performance of a heat sink microchannel working with biologically produced silver-water nanofluid: Experimental assessment. Exp. Therm. Fluid Sci..

[13] Sarafraz M (2017). On the convective thermal performance of a CPU cooler working with liquid gallium and CuO/water nanofluid: A comparative study. Appl. Therm. Eng..

[14] Sarafraz M, Hormozi F (2015). Intensification of forced convection heat transfer using biological nanofluid in a double-pipe heat exchanger. Exp. Therm. Fluid Sci..

[15] Sarafraz M, Hormozi F (2016). Heat transfer, pressure drop and fouling studies of multi-walled carbon nanotube nano-fluids inside a plate heat exchanger. Exp. Therm. Fluid Sci..

[16] Sarafraz M, Hormozi F, Kamalgharibi M (2014). Sedimentation and convective boiling heat transfer of CuO-water/ethylene glycol nanofluids. Heat Mass Transf..

[17] Sarafraz MM, Hormozi F (2014). forced convective and nucleate flow boiling heat transfer to alumnia nanofluids. Period. Polytech. Chem. Eng..

[18] Lahari, M. C., *et al.* Investigation on heat transfer properties of water based TiO_2_–ZnO nanofluids. *In IOP Conference Series: Materials Science and Engineering*. (IOP Publishing, 2018).

[19] Daghigh R, Zandi P (2018). Experimental analysis of heat transfer in spiral coils using nanofluids and coil geometry change in a solar system. Appl. Therm. Eng..

[20] Ghalib, L., *et al.* Flow and Heat transfer experimental investigation of nanofluid in a double pipe heat exchanger. In *IOP Conference Series: Materials Science and Engineering.* (IOP Publishing, 2018).

[21] Tariq, R., Sheikh, K. A., Shayyan, M. Study of thermal performance of common heat exchangers by using nanofluids. In *2018 International Conference on Applied and Engineering Mathematics (ICAEM)* (IEEE, 2018).

[22] Haq RU (2018). Heat exchange within the partially heated C-shape cavity filled with the water based SWCNTs. Int. J. Heat Mass Transf..

[23] Wang Z, Wu Z, Sundén B (2018). Effects of graphene ethylene glycol/water nanofluids on the performance of a brazed plate heat exchanger. J. Nanofluids.

[24] Huminic G, Huminic A (2018). Hybrid nanofluids for heat transfer applications—A state-of-the-art review. Int. J. Heat Mass Transf..

[25] Nakhchi ME, Esfahani JA (2018). Cu-water nanofluid flow and heat transfer in a heat exchanger tube equipped with cross-cut twisted tape. Powder Technol..

[26] Raei B, Peyghambarzadeh S, Asl RS (2018). Experimental investigation on heat transfer and flow resistance of drag-reducing alumina nanofluid in a fin-and-tube heat exchanger. Appl. Therm. Eng..

[27] Shankar MS, Immanuel P, Eswaran M (2018). Heat transfer analysis of shell and tube heat exchanger using Al_2_O_3_ nanofluids. Int. J. Mech. Eng. Technol. IJMET.

[28] Vandrangi SK (2018). Friction factor analysis of SiO_2_ and Al_2_O_3_ nanofluids dispersed in 60 egw and 40 egw base fluids. J. Adv. Res. Fluid Mech. Therm. Sci..

[29] Han D, He W, Asif F (2017). Experimental study of heat transfer enhancement using nanofluid in double tube heat exchanger. Energy Proc..

[30] Bahiraei M, Godini A, Shahsavar A (2018). Thermal and hydraulic characteristics of a minichannel heat exchanger operated with a non-Newtonian hybrid nanofluid. J. Taiwan Inst. Chem. Eng..

[31] Naik BAK, Vinod AV (2018). Heat transfer enhancement using non-Newtonian nanofluids in a shell and helical coil heat exchanger. Exp. Therm. Fluid Sci..

[32] Kumar V, Tiwari AK, Ghosh SK (2017). Characterization and performance of nanofluids in plate heat exchanger. Mater. Today Proc..

[33] Zhang C (2017). Investigation of flow boiling performance and the resulting surface deposition of graphene oxide nanofluid in microchannels. Exp. Therm. Fluid Sci..

[34] Das SK, Putra N, Roetzel W (2003). Pool boiling of nano-fluids on horizontal narrow tubes. Int. J. Multiphase Flow.

[35] Anoop K, Cox J, Sadr R (2013). Thermal evaluation of nanofluids in heat exchangers. Int. Commun. Heat Mass Transf..

[36] Maayah, E. S., Duwairi, H. M., Maayah, B. S. Analytical model of solar energy storage using non-Newtonian Fluid in a saturated porous media in fully developed region: Carboxymethyl cellulose (CMC) and graphite model. *AIMS Energy*. **9**, 213 (2021).

[37] Yunus, A. Ç. *Heat and Mass Transfer: Fundamentals and Applications* (McGraw-Hill Education, 2019).

[38] Rao VN, Sankar BR (2019). Heat transfer and friction factor investigations of CuO nanofluid flow in a double pipe U-bend heat exchanger. Mater. Today Proc..

[39] Maayah ES, Duwairi HM, Maayah B (2021). Analytical model of solar energy storage using non-Newtonian Fluid in a saturated porous media in fully developed region: carboxymethyl cellulose (CMC) and graphite model. AIMS Energy.

[40] Gnielinski V (1976). New equations for heat and mass transfer in turbulent pipe and channel flow. Int. Chem. Eng..

[41] Duangthongsuk W, Wong WS (2009). Heat transfer enhancement and pressure drop characteristics of TiO_2_—Water nanofluid in a double-tube counter flow heat exchanger. Int. J. Heat Mass Transf..

